# Gestion des déchets ménagers dans l’aire de santé Bulaska à Mbuji-Mayi en République Démocratique du Congo

**DOI:** 10.11604/pamj.2016.24.252.9262

**Published:** 2016-07-19

**Authors:** Kasangye Kangoy, John Ngoyi, Olive Mudimbiyi

**Affiliations:** 1 Université de Kabinda, Ecole de Santé Publique, Kabinda, Kasaï Oriental, République Démocratique du Congo (RDC); 2 Shandong University, School of Public Health, Social Medicine and Health Management Department, Jinan, Shandong, China

**Keywords:** Gestion, déchet, ménage, Management, waste, household, health area

## Abstract

**Introduction:**

La présence des déchets ménagers dans les voies publiques a une influence sur l’hygiène de l’environnement, ils entrainent l’insalubrité et peuvent être facteurs des certaines maladies dont quelques-unes peuvent être épidémiques. Au cours des deux dernières décennies, la question de la gestion des déchets est devenue de plus en plus complexe autant pour les pays développés que ceux sous-développés. L’objectif de cette étude était de déterminer les types de déchets et le mode de gestion des déchets génère par les ménages.

**Méthodes:**

Cette étude est descriptive transversale, réalisée dans l’aire de sante Bulaska, Kasaï oriental, c’est une approche prospective appuyée par l’interview et l’observation active. Le questionnaire a été adressé au responsable du ménage ou au délègue, du 21 au 25 juin 2010, sur 170 ménages ce qui constituent un échantillon de convenance.

**Résultats:**

Cette étude a révélé ce qui suit: 94,7% des enquêtes qui avaient répondu a notre questionnaire étaient de sexe féminin; 47% des enquêtes avaient un niveau d’étude primaire; 41,1% des enquêtes étaient des ménagères; la taille médiane de ménage était de 7 personnes par ménage; dans 83,5% des cas les déchets génères étaient solides; 50% des ménages de l’aire de sante utilisent la voie publique comme poubelle.

**Conclusion:**

Eu égard au résultat de cette étude, développer plus les programmes de sensibilisation sur l’assainissement de l’environnement s’avère nécessaire.

## Abstract (en)

**Introduction:**

The presence of household waste on public roads affects environmental health leading to unsanitary conditions which may cause disease outbreaks, some of which may occur in epidemic form. Over the past two decades, waste management has become increasingly complex both for developing and underdeveloping countries. This study aims to determine the types of waste and the management of waste generated by the households.

**Methods:**

This descriptive cross-sectional study conducted in the health district of Bulaska, Kasai Oriental, is a forward-looking approach based upon interview and active observation. The questionnaire was addressed to the head of household or the delegate out of 170 households, representing a convenience sample, from 21 to 25 June 2010.

**Results:**

This study revealed that: 94.7% of respondents who answered our questionnaire were female; 47% of respondents had a primary level of study; 41.1% of respondents were housewives; the average household size was 7 people per household; in 83.5% of cases the wastes generated were solid. 50% of households in the health area used public road for trash disposal.

**Conclusion:**

Given the results of this study, further development of awareness programs on environmental sanitation is necessary.

## Introduction

Notre étude porte sur la gestion des déchets ménagers dans l’aire de santé Bulaska dans la zone de sante de Kansele, ville de Mbujimayi. Au cours des deux dernières décennies, la question de la gestion des déchets est devenue de plus en plus complexe autant pour les pays du Nord que pour ceux du Sud. Depuis la mondialisation des échanges, des techniques et des idées, place la question des déchets dans la liste de préoccupation ménagère des sociétés dans ce troisième millénaire [1]. A ce titre, la problématique de la gestion des déchets s’insère dans un contexte plus global de développement durable et est soumis à des enjeux économiques, sociaux et environnementaux. Les déchets non contrôles sont une source de prolifération d’insectes, des accidents d’enfants et des toxines spécifiques [2]. L’étude menée sur la gestion des déchets d’élevage et ménagère biodégradable au Niger a montré que dans 14,25% de cas les déchets étaient jetés dans la rivière, dans 15,83% les déchets étaient entasses dans les coins des parcelles, dans 22,43% des cas les déchets étaient utilises comme engrain dans les champs ou jardins, et dans 30,34% des cas les déchets étaient incinérés à ciel ouvert [3]. L’urbanisation des pays d’Afrique a causé la détérioration de l’environnement. L’une de conséquences les plus inquiétantes dans le monde en développement et particulièrement en Afrique, réside pourtant dans les problèmes des gestions des déchets solides, liquides et toxiques. Dans les pays en voie de développement, l’enlèvement des déchets ménagers n’est assuré que dans quelques zones résidentielles et dans les restes des villes, les déchets sont déposés sur la route ou dans des sites illégaux, ou sont déversés dans les égouts ou enterres dans des charges à ciel ouvert [4, 5]. La présence des déchets ménagers dans les voies publiques a une influence sur l’hygiène de l’environnement, ils entrainent l’insalubrité et peuvent être facteurs des certaines maladies dont quelques-unes peuvent être épidémiques, hébergent des mouches, d’insectes cafards, moustiques, dégageant les odeurs malsaine. Dans la ville de Mbuji-Mayi, les rues constituent les lieux privilégiés ou sont déversés les déchets, le péril fécal reste mal géré du fait de manque des installations hygiéniques. Voilà ce qui entraine la persistance des maladies dites des mains sales. En 2003, la ville avait connu une épidémie de choléra de triste mémoire et toutes les tentatives d’organisation des campagnes d’assainissement de la ville se sont terminées par un échec. Notre étude descriptive transversale a été réalisée dans l’aire de sante Bulaska, zone de sante de Kansele, ville de Mbuji Mayi, Province du Kasaï Oriental.

## Méthodes

Pour atteindre nos objectifs nous avons fait recours à une approche prospective appuyée par l’interview et l’observation active. Les données relatives à la réalisation de ce travail ont été collectées pendant cinq jours soit du 21 au 25 juin 2010 et les enquêtes ce sont déroulées du 21 au 25 juin 2010, et les enquêtes ce sont déroulées dans 170 ménages qui constituent un échantillon de convenance. Le questionnaire s’adresse au responsable du ménage ou on délègue. La visite de la parcelle et l’interview étaient des principales techniques de collecte données.

## Résultats

### Données socio-démographiques

**Figure 1:**
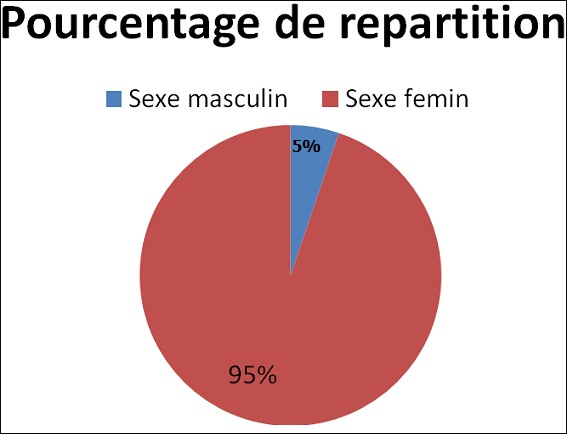
Répartition des cas selon le sexe

La Figure 1 nous a renseigné que les enquêtes du sexe féminin était plus représentées (94,7%) par rapport à celles du sexe masculin (5.20%). La lecture du Tableau 1 nous montre que 47,6% des enquêtes étaient du niveau primaire, 30% étaient du niveau secondaire, 14,1% étaient sans niveau alors que le niveau supérieur et universitaire n’ont représenté que 8,2%.

**Tableau 1:** Répartition des cas selon le sexe

Niveau d’instruction	Cas	%
Sans niveau	24	14,1
Primaire	81	47,6
Secondaire	51	30
Supérieur et universitaire	14	8,2
Total	170	100

### Données en rapport l’environnement

**Figure 2:**
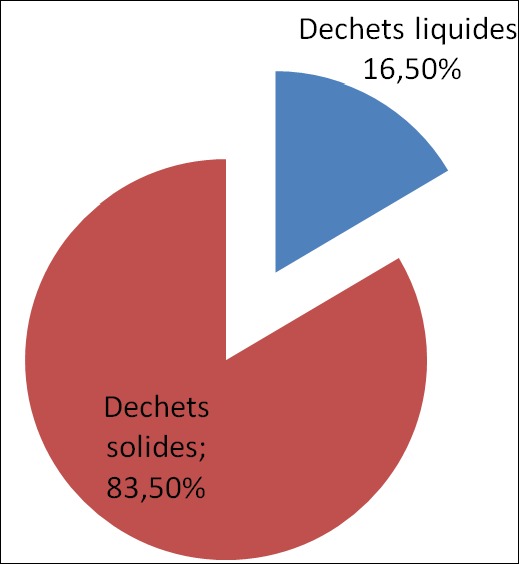
Répartition des déchets selon qu’ils sont solides ou liquides

A la lumière de la Figure 2, nous avons remarqué que dans 83.5% des cas, les déchets étaient solides et 16,5% étaient liquides. La lecture du Tableau 2, nous a renseigne que dans 68,2% des cas les déchets étaient constitués des eaux usées, des restes alimentaires, des balayures…et dans 28% étaient constitués des matières usées, déconsidérées (habits usées…). La lecture du Tableau 3, nous a montré que dans les enquêtes, 50% des cas jetaient les déchets sur la voie publique, 28,8% des cas les jetaient dans les trous à ordures et 16,4% des cas procédaient à l’incinération à ciel ouvert.

**Tableau 2:** Répartition de cas selon le niveau d’instruction

Composition des déchets ménagers	Cas	%
Matières usées déconsidérées	49	28,8
Eaux usées, restes alimentaires, balayures…	116	68,2
Matières fécales	5	2,9
**Total**	**170**	**100**

**Tableau 3:** Répartition des déchets selon le mode d’évacuation

Mode de gestion/ évacuation	cas	%
Voie publique	85	50
Trou a ordure	49	28,8
Enfouissement	3	1,7
Incinération à ciel ouvert	28	16,4
Ravin	5	2,9
**Total**	**170**	**100**

## Discussion

La mise en œuvre d’un programme d’assainissement du milieu passe par les études des états des lieux dans les entités concernées par ce programme. A l’heure actuelle l’urbanisation rapide et sauvage des pays d’Afrique a causé la détérioration de l’environnement. L’une des conséquences les plus inquiétantes dans le monde en développement et particulièrement en Afrique, réside d’ailleurs dans les problèmes des gestions de déchets solides, liquides et toxiques. Des incidents qui ont eu lieu récemment dans les grands centres urbains d’Afrique montrent que le problème de la gestion des déchets a atteint des proportions telles que les mesures prises par différents niveaux d’administration et les spécialistes se sont révélées infructueuses [6, 7]. Notre étude a montré qu’en rapport avec le niveau d’instruction 14,1% de nos enquêtes n’avaient aucun niveau d’instruction 14,1% de nos enquêtes n’avaient aucun niveau d’instruction, 47,6% du niveau primaire (Tableau 2). Ceci confirme le rapport de l’Unicef sur le faible taux de scolarité dans la province de Kasaï oriental [8]. Par rapport au sexe, nous avons retenu que ces sont les femmes qui avaient répondus nombreuses (94,7%) par rapport aux hommes (5.2%) (Figure 1). Ceci peut s’expliquer dans le sens que la plupart des hommes ne s’occupent pas de la gestion des déchets générés par leurs ménages laissant cette tache a la femme seule. En ce qui concerne les types des déchets nous avons noté que dans 83,5% des cas de déchets générés étaient solides alors que 16,5% des cas les déchets étaient liquide (Figure 2). Considérant la composition des déchets notre étude a montré que dans 68,2% des cas les déchets étaient générés dans la chaine de préparation des aliments; dans 28,8% les déchets étaient composés des matières usées déconsidérées telles que les habits, les sachets, les casseroles troues, les boites de conserves…le fait que les eaux usées et autres occupent une première place s’explique par le fait que l’eau est la denrée la plus sollicitée dans les travaux de ménage. En rapport avec l’évacuation des déchets, nous avons noté que la population de notre aire de sante utilisait la voie publique dans 50% des cas, suivi de trou à ordure dans 28,8%. L’incinération à ciel ouvert était utilisée dans 16,4% de cas. Par rapport à l’incinération, nos résultats diffèrent avec ceux de l’étude menée au Niger sur la gestion des déchets d’élevage et ménager biodégradable. D’après cette étude, l’incinération à ciel ouvert était pratiquée dans 16,4% des cas [3].

## Conclusion

La bonne gestion des déchets ménagers est un élément important de l’assainissement de l’environnement et la mauvaise gestion peut être un facteur favorisant la propagation des nombreuses maladies. Toutes les actions préventives qui visent la promotion de la sante ne peuvent être efficaces que si elles sont menées dans un environnement compatible avec la vie saine. L’étude menée selon la démarche prospective transversale nous a conduit aux observations ci-après: La gestion des déchets concerne plus les femmes (94,7%) que les hommes; Les lieux publics restent les endroits privilégiés ou 50% de ménages jettent leurs déchets; Dans 83,5% des cas les déchets générés étaient solides. Eu égard a ce qui précède, nous pensons que les décideurs, les organisations non gouvernementales peuvent capitaliser les données de cette études pour développer les programmes de sensibilisation de la population, qui est exposée en vivant dans un environnement insalubre.

### Etat des connaissances actuelles sur le sujet

- La gestion de déchets ménagères restent un problème globale tant pour les pays développés que pour ceux en voie de développement;
- Les déchets non contrôlés sont une source de prolifération d’insectes, des accidents d’enfants et des toxines spécifiques;
- Ainsi La bonne gestion des déchets ménagers est un élément important de l’assainissement de l’environnement et sa mauvaise gestion peut être un facteur favorisant la propagation des nombreuses maladies.

### Contribution de notre étude à la connaissance

- Les lieux publics restent les endroits privilégiés ou les ménages jettent leurs déchets dans les pays sous-développés comme la RDC;
- Etant donné que la gestion des déchets ménagers concerne plus les femmes que les hommes, une sensibilisation sur l’assainissement du milieu tournée plus sur les femmes s’avère nécessaire;
- Les déchets générés par les ménages sont plus solides que liquides, cette étude donne donc quelques recommandations pour instruire la population sur la gestion spécialisée de déchets solides.
